# The global prevalence of Daptomycin, Tigecycline, Quinupristin/Dalfopristin, and Linezolid-resistant *Staphylococcus aureus* and coagulase–negative staphylococci strains: a systematic review and meta-analysis

**DOI:** 10.1186/s13756-020-00714-9

**Published:** 2020-04-22

**Authors:** Aref Shariati, Masoud Dadashi, Zahra Chegini, Alex van Belkum, Mehdi Mirzaii, Seyed Sajjad Khoramrooz, Davood Darban-Sarokhalil

**Affiliations:** 1 Student Research Committee, Department of Microbiology, School of Medicine, Iran University of Medical Sciences, Tehran, Iran; 2 Department of Microbiology and Immunology, School of Medicine, Alborz University of Medical Sciences, Karaj, Iran; 3 Open Innovation & Partnerships, Route de Port Michaud, 38390, La Balme Les Grottes, France; 4 School of Medicine, Shahroud University of Medical Sciences, Shahroud, Iran; 5 Cellular and Molecular Research Center and Department of Microbiology, School of Medicine, Yasuj University of Medical Sciences, Yasuj, Iran; 6 Department of Microbiology, School of Medicine, Iran University of Medical Sciences, Tehran, Iran

**Keywords:** Linezolid, Daptomycin, Tigecycline, Quinupristin/Dalfopristin, Synercid, Meta-analysis, *S. aureus*, MRSA, CoNS

## Abstract

**Objective:**

Methicillin-resistant *Staphylococcus aureus* (MRSA) and methicillin-resistant coagulase-negative Staphylococcus (MRCoNS) are among the main causes of nosocomial infections, which have caused major problems in recent years due to continuously increasing spread of various antibiotic resistance features. Apparently, vancomycin is still an effective antibiotic for treatment of infections caused by these bacteria but in recent years, additional resistance phenotypes have led to the accelerated introduction of newer agents such as linezolid, tigecycline, daptomycin, and quinupristin/dalfopristin (Q/D). Due to limited data availability on the global rate of resistance to these antibiotics, in the present study, the resistance rates of *S. aureus*, Methicillin-resistant *S. aureus* (MRSA), and CoNS to these antibiotics were collected.

**Method:**

Several databases including web of science, EMBASE, and Medline (via PubMed), were searched (September 2018) to identify those studies that address MRSA, and CONS resistance to linezolid, tigecycline, daptomycin, and Q/D around the world.

**Result:**

Most studies that reported resistant staphylococci were from the United States, Canada, and the European continent, while African and Asian countries reported the least resistance to these antibiotics. Our results showed that linezolid had the best inhibitory effect on *S. aureus.* Although resistances to this antibiotic have been reported from different countries, however, due to the high volume of the samples and the low number of resistance, in terms of statistical analyzes, the resistance to this antibiotic is zero*.* Moreover, linezolid, daptomycin and tigecycline effectively (99.9%) inhibit MRSA. Studies have shown that CoNS with 0.3% show the lowest resistance to linezolid and daptomycin, while analyzes introduced tigecycline with 1.6% resistance as the least effective antibiotic for these bacteria. Finally, MRSA and CoNS had a greater resistance to Q/D with 0.7 and 0.6%, respectively and due to its significant side effects and drug-drug interactions; it appears that its use is subject to limitations.

**Conclusion:**

The present study shows that resistance to new agents is low in staphylococci and these antibiotics can still be used for treatment of staphylococcal infections in the world.

## Introduction

Methicillin-resistant *Staphylococcus aureus* (MRSA) and methicillin-resistant coagulase-negative staphylococci (MRCoNS) represent main causes of hospital- and community-acquired infections; because of their increasing numbers and elevated mortality, morbidity, and medical expenses, they have become a global concern in recent years [1, 2]. Staphylococci contain virulence factors and toxins that cause various diseases including blood, skin and soft tissues infections, nosocomial infections connected with the presence of medical devices, and toxic shock syndrome [3]. The *mecA* gene, located in the SCCmec region, is responsible for the expression of methicillin resistance through PBP2a—an altered penicillin-binding protein that is characterized by its low affinity to penicillin and other beta-lactam drugs [4]. For both MRSA and MRCoNS vancomycin is used as the first line drug for treatment. However, in recent years, decreased susceptibility and even resistance to vancomycin and other antibiotics, including aminoglycosides, tetracyclines, and lincosamides, have been reported in many parts of the world [5–7]. Therefore, for the treatment of severe infections caused by multi-drug resistant staphylococci, new antibiotics such as daptomycin, linezolid, tigecycline, and Quinupristin/Dalfopristin (Q/D) were introduced [8]. Daptomycin, a cyclic lipopeptide antibiotic, is the second most important anti-MRSA drug, which received FDA approval in 2003 and approval by the European Medicines Agency (EMA) in 2005. It is mostly used for the treatment of acute bacterial skin and soft tissues infections [9]. Daptomycin is still quite active against staphylococci and enterococci; however, resistance to this antibiotic has been reported over the past years due to mutation of various genes (*dltABCD* genes, *mprF* and *rpoB*), causing changes in membrane fluidity, cell wall thickness, and membrane charge [10, 11]. Tigecycline is an example of a new class of broad-spectrum antimicrobial agents known as glycylcyclines with activity against Gram-positive and Gram-negative organisms. This antibiotic was approved by FDA (2005–2009) for the treatment of skin infections, intra-abdominal infections and community-acquired bacterial pneumonia [12, 13]. Tigecycline provides an alternative treatment for complicated MRSA and vancomycin resistant enterococci (VRE) infections; due to mutations in *mep*R and *mep*A genes that result in overexpression of efflux pumps, resistant phenotypes have been reported in recent studies [13]. Linezolid is another new antibiotic that was approved in 2000 for the treatment of MRSA and MRCoNS infections and infections caused by VRE. Linezolid binds to the 50S ribosomal subunit of the 23S rRNA molecule and inhibits protein synthesis. *Cfr* gene encodes a methyltransferase that modifies the 23S rRNA site of the 50S ribosomal subunit and prevents linezolid from binding to it [14]. Q/D is composed of two streptogramins (70% dalfopristin (streptogramin A) and 30% quinupristin (streptogramin B)), which was approved in 1999 as a treatment option for VRE and MRSA infections. This drug consists of quinupristin that inhibits late-stage protein synthesis, while dalfopristin inhibits early-stage protein synthesis. It should be noted that, Synercid® (formerly RP59000; Rhone-Poulenc) is the first semisynthetic injectable streptogramin and it is used as a trade name for Q/D [15, 16]. The World Health Organization (WHO) has considered MRSA as important antibiotic-resistant bacteria and put them on their priority list. All organisms on that list require new treatment modalities and substantiate an urgent overall need for new antimicrobial drugs [17]. According to the authors’ knowledge, no comprehensive data are available on the resistance levels to daptomycin, Q/D, linezolid, and tigecycline among MRSA and MRCoNS strains. This study aims to investigate the prevalence of resistance to the mentioned antibiotics among staphylococcal strains isolated from clinical samples around the world.

## Methods

**Fig. 1:**
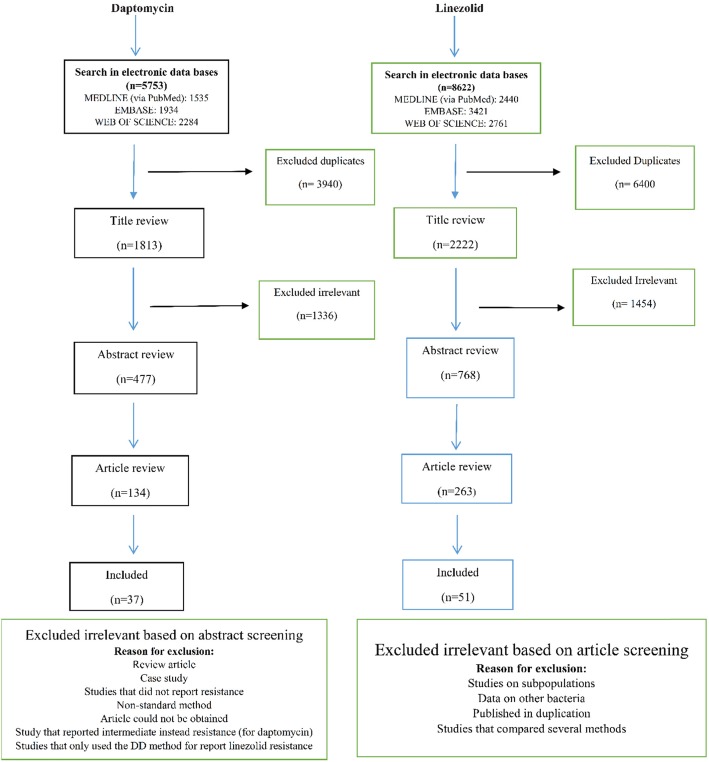
Flow chart detailing review process and study selection for linezolid and daptomycin

**Fig. 2:**
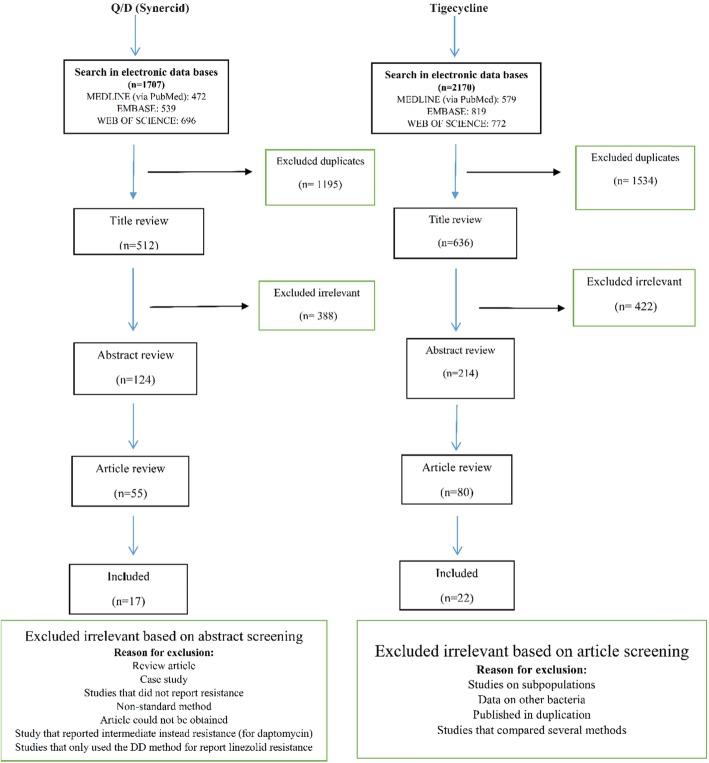
Flow chart detailing review process and study selection for Q/D and tigecycline

We conducted a literature search through databases, including web of science, EMBASE, and Medline (via PubMed), using the versions of September 2018. The historic publication year was unrestricted and the search was limited to original articles. The following search keywords were obtained from the National Library of Medicine’s medical subject heading (MeSH) terms or titles or abstracts with the help of Boolean operators (and, or): “staph”, “staphylococcus”, “staphylococci”, “staphylococcal”, “staphylococcaceae” and “Linezolid”, “Daptomycin”, “Tigecycline”, “Quinupristin/Dalfopristin”, and “Synercid”. Two independent reviewers screened the titles and abstracts of original articles and posters; if an article appeared relevant (Figs. 1 and 2), the full text was reviewed. We used the Clinical and Laboratory Standards Institute (CLSI) and the European Committee on Antimicrobial Susceptibility Testing (EUCAST) for daptomycin, linezolid, Q/D resistance and tigecycline resistance in Staphylococci, respectively (there is no standard for tigecycline in staphylococci in the CLSI). The resistance cut-off rates are defined in the following ranges ≤1 mg/L, ≥8 mg/L, ≥4 mg/L, and > 5 mg/L, respectively. We considered all articles that evaluated antibiotic resistance by different methods such as broth microdilution (BMD), agar dilution, disk diffusion (DD), E-test and Vitek or Vitek 2 or any other automated instruments. It should be noted that, the final version of the CLSI (2018) states that staphylococci with resistant results to linezolid by DD should be confirmed by using an MIC method, therefore, studies that only used the DD method for susceptibility to the linezolid were excluded. Moreover, case reports, basic research on the resistance mechanism of the mentioned antibiotics, and review articles were excluded from this study.

### Meta-analysis

#### Quality assessment

All reviewed studies were subjected to a quality assessment (designed by the Joanna Briggs Institute) and only high-quality investigations were evaluated in our final analysis [18–116].

#### Data analysis

The analysis was performed by STATA (version 14.0) software. The data were pooled using a fixed effects model (FEM) [117] and a random effects model (REM) [118]. Statistical heterogeneity was assessed by statistical methods [119] and was evaluated using the Q-test and the I2 statistical methods [118]. *P*-value < 0.1 was regarded as statistically significant [120].

## Results

**Fig. 3:**
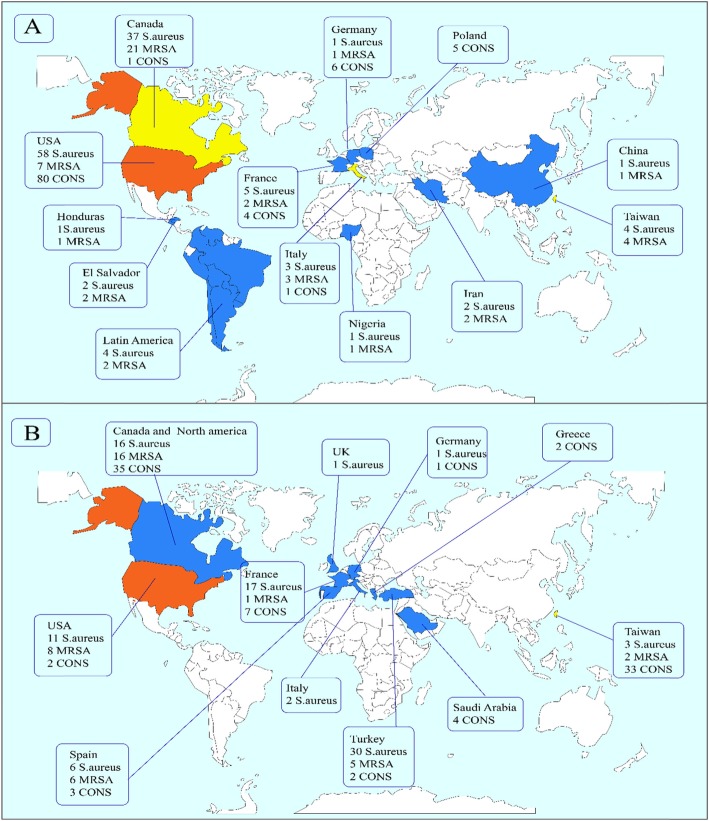
The global prevalence of **a**) Tigecycline, **b**) Quinupristin/Dalfopristin-resistant *S. aureus*, MRSA and CoNS

**Fig. 4:**
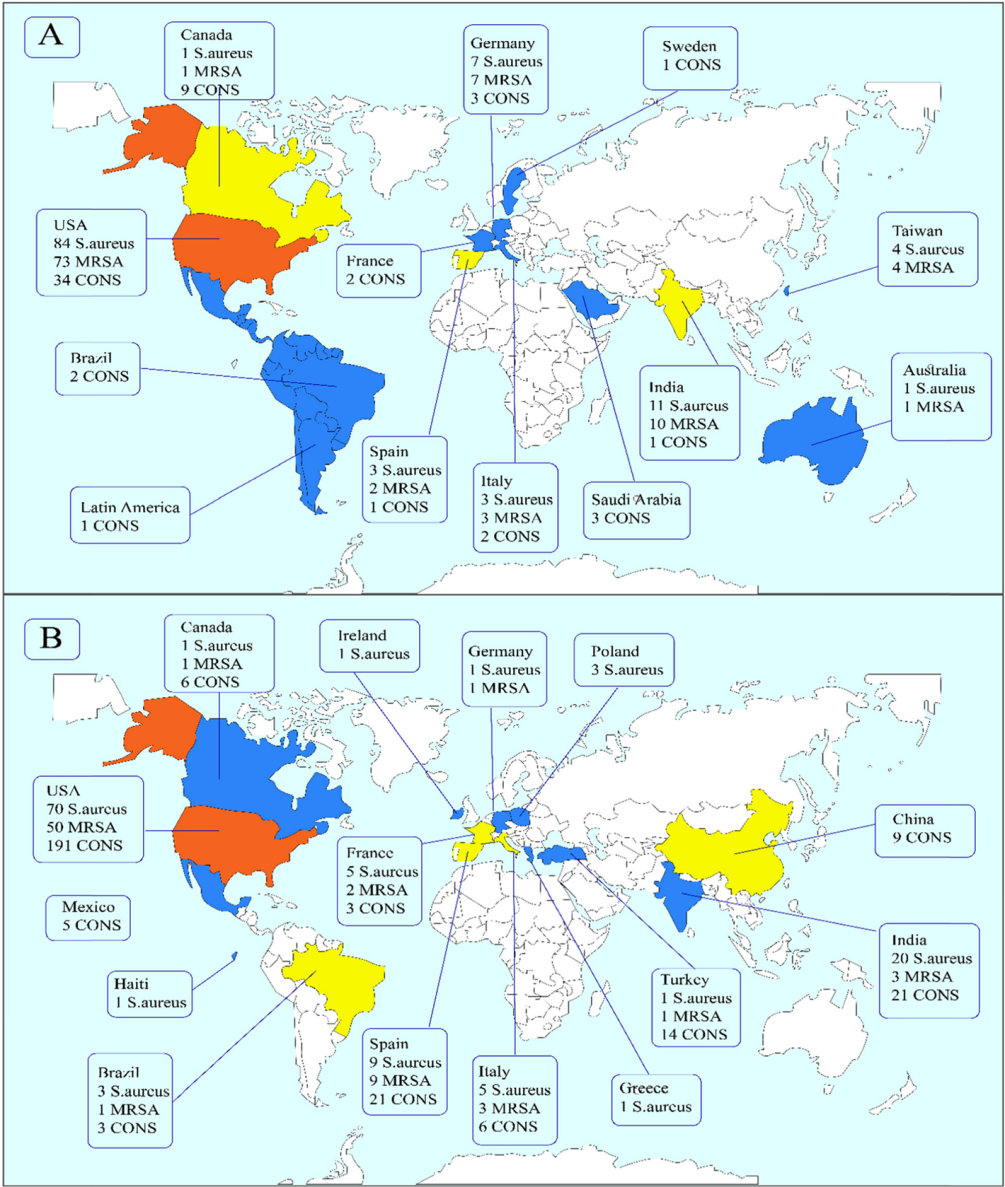
The global prevalence of a) Daptomycin and b) linezolid-resistant *S. aureus*, MRSA and CoNS

This study identified 1813, 2222, 512, and 636 articles for daptomycin, linezolid, Q/D (Synercid), and tigecycline, respectively, in the first step. Then, upon secondary screening, a large number of articles were excluded on the basis of title and abstract evaluation because of the lack of relevance to the study principles, and the reasons for the deletion of these articles are presented in Figs. 1 and 2. Therefore, 477, 768, 124, and 214 articles for the mentioned antibiotics were reviewed with full text, and a number of papers were excluded from the study for the reasons listed in Figs. 1 and 2. Finally, 37, 51, 17, and 22 eligible studies for daptomycin, linezolid, Q/D, and tigecycline were chosen for final analysis, respectively. Resistance percentage in *S. aureus*, MRSA and CONS to the mentioned antibiotics is shown in Table 1. The characteristics of the included articles are summarized in Tables 2, 3, 4 and 5. All pertinent studies were included from around the world (25 different countries) (Tables 2, 3, 4 and 5). The USA was the most frequently represented country for all antibiotics followed by Canada and European countries (Italy and Spain). From the African continent, only one study from Nigeria, where tigecycline resistance in one isolate was reported (Fig. 3). Linezolid-resistant staphylococci from 15 countries were included in the present study, which was more widely distributed among antibiotics (Fig. 4). Strains were isolated from various clinical samples including blood, wound, skin, urine, respiratory tract, sputum, catheter, bone, etc. A majority of studies used BMD, E-test, agar dilution, disk diffusion, and Vitek or vitek 2. Our results showed that linezolid had the best inhibitory effect on *S. aureus.* Although resistance to the linezolid has been reported from different countries, due to the high volume of the samples and the low number of resistance, in terms of statistical analyzes, the resistance to this antibiotic is zero*.* Moreover, linezolid and tigecycline effectively (99.9%) inhibit MRSA (Table 1). Studies have shown that CoNS with 0.3% show the lowest resistance to linezolid and daptomycin, while analyzes introduced tigecycline with 1.6% resistance as the least effective antibiotic for these bacteria. Finally, MRSA and CoNS had a greater resistance to Q/D with 0.7 and 0.6%, respectively.

**Table 1:** Resistance percentages in *S. aureus*, MRSA and CoNS to different antibiotics

***S. aureus***				
	Linezolid	Daptomycin	Tigecycline	Q/D
Resistance rate (%)	0.0%[CI% (0.0–0.0)]	0.1 [CI% (0.1–0.1)]	0.1 [CI% (0–0.1)]	0.1 [CI% (0.1–0.2)]
p-value	0.04	0.02	0.09	0.88
**MRSA**				
Resistance rate (%)	0.1 [CI% (0–0.1)]	0.1 [CI% (0.1–0.1)]	0.1 [CI% (0–0.1)]	0.7 [CI% (0.3–1)]
p-value	0.33	0.00	0.00	0.00
**CoNS**				
Resistance rate (%)	0.3 [CI% (0.2–0.4)]	0.3 [CI% (0.2–0.4)]	1.6 [CI% (1.2–1.9)]	0.6 [CI% (0.3–0.9)]
p-value	0.04	0.37	0.00	0.00

MRSA; Methicillin-resistant *Staphylococcus aureus*, CoNS; Coagulase-negative staphylococci, Q/D; Quinupristin / Dalfopristin

**Table 2:** Characteristics of the articles that were included in the meta-analysis and reported resistance to tigecycline

First name	Time of study	Published time	Country	Total staphylococcus	*S. aureus*	MRSA	CoNS	*S. aureus* Tigecycline-Resistant	MRSA Tigecycline- Resistant	CoNS Tigecycline -Resistant	Susceptibility testing method	Isolation source
Morrissey [76]	2011	2012	Germany	81	43	43	38	1	1	6	BMD	Bacteraemia and Skin infection
Ayepola [20]		2015	Nigeria	209	209	6		1	1		Automated VITEK-2 system	Clinical specimens
Garza-González E [48]	2009	2013	Honduras	61	61	21		1	1		BMD	Urine, Blood, Respiratory tract, Skin, Wound, Body fluid
Garza-González E [48]	2009	2013	El Salvador	34	34	19		2	2		BMD	Urine, Blood, Respiratory tract, Skin, Wound, Body fluid
Xi [112]	2014–2016	2018	China	15	13	11	2	1	1		DD	Clinical specimens
Wang [111]	2006–2010	2015	Taiwan	670	670	670		3	3		Automated VITEK-2 system	Blood infection
Adam [18]	2007–2011	2013	Canada	4177	4177	1266		6	3		BMD	Blood, Respiratory tract, Urine, Wound
Cassettari [28]	2010–2011	2011	Italy	280	201	102	79	1	1		BMD	Skin and soft tissue infections, Hospital-acquired pneumonia
Bongiorno [25]	2012	2018	Italy	50	50	50		2	2		BMD	Lower respiratory tract infections, Skin and soft-tissue, Blood
Zhanel [114]	2007–2011	2013	Canada	6623	5443	2500	1180	8	4		BMD	Wound, Urinary tract, Blood
Flamm [40]	2010	2012	USA	4049	3105	1578	944	2	1		BMD	Blood, Pneumonia, Skin
Flamm [41]	2013	2015	USA	3433	3035	1454	398	1	1		BMD	Blood, Skin, Soft tissue
Yousefi [113]	2014–2015	2017	Iran	54	54	54		2	2		BMD	UTI
Hodile [51]	2010–2014	2017	France	440	440	325		5	2		BMD	Bronchopulmonary infections
Chen [30]	2006–2010	2014	Taiwan	1725	1725	1725		1	1		BMD	Blood, Pus
Zhanel [115]	2007–2009	2011	Canada	3910	3589	889	321	5		1	BMD	Wound, Urinary tract, Blood, Respiratory tract
Vega [110]	2004–2015	2017	Latin America	4563	4563	2202		4	2		BMD	Clinical specimens
Sader [93]	2006–2012	2014	USA	28,278	28,278	14,756		2	2		BMD	Blood, Wound, Skin, Pneumonia
Putnam [86]	2004–2008	2010	USA	18,917	18,917	10,242		3	3		BMD	Skin, Intra-abdominal, Bacteraemia
Karlowsky [65]	2011–2015	2017	Canada	3760	3408	728		18	14		BMD	Urine, Blood, Respiratory tract, Skin, Wound, Body fluid
Morrissey [76]	2011	2012	Italy	82	41	41	41			1	BMD	Bacteraemia, Skin infection
Brzychczy-wolch [26]	2009	2013	Poland	100			100			5	DD	Blood, Pneumonia
Jan [56]	2006–2009	2012	France	216	26	6	190			4	Agar dilution	Implantable cardioverter defibrillator infection
Sader [101]	2000–2004	2005	USA	12,335	8765	3050	3570	50		80	BMD	Blood

Abbreviations: DD; disk diffusion, BMD; broth microdilution

**Table 3:** Characteristics of the articles that were included in the meta-analysis and reported resistance to Q/D

First name	Time of study	Published time	Country	Total staphylococcus	*S. aureus*	MRSA	CoNS	*S. aureus* Q/D-Resistant	MRSA Q/D- Resistant	CoNS Q/D-Resistant	Susceptibility testing method	Isolation source
Petrelli [79]	2003–2004	2007	Italy	37	37	16		1			DD	Blood infection
McDonald [72]	1998–2000	2004	Taiwan	554	400	240	154	1		1	BMD	Blood, Urine, Wound, Respiratory tract
Luh [69]	1996–1999	2000	Taiwan	554	149	80	405	1	1	32	Agar dilution	Blood, Respiratory tract, Cerebrospinal fluid, Bile, Wound, Rectal swab
Picazo [85]	2010	2011	Spain	702	503	187	199	1	1	3	BMD	Medical canters
Sader [103]	2002–2004	2006	Germany	1232	715		517	1		1	BMD	Skin infection, Blood
Sader [103]	2002–2004	2006	Italy	685	386		299	1			BMD	Skin infection, Blood
Sader [103]	2002–2004	2006	UK	593	531		62	1			BMD	Skin infection, Blood
Draghi [36]	2004	2005	USA	3368	2872	1556	496	2			BMD	Skin, Blood, Respiratory tract
Ballow [21]		2002	North America	11,671	7038	2721	4633	10	10	20	BMD	Medical canters
Decousser [34]	2000	2003	France	364	242	87	122	1	1		E-test	Blood
Hsueh [52]	1991–2003	2005	Taiwan	100	100	100		1	1		Agar dilution	Clinical specimens
Limoncu [68]		2003	Turkey	149	149	52		30	5		BMD	Clinical specimens
Jones [59]	1996–1997	2001	USA	1778	1290	623	488	7	6	1	DD	Wound, Abdominal cavity, Respiratory tract, Urinary tract, Blood
Anastasiou [19]	2001–2003	2008	North America	360	360	360		6	6		BMD	Hospital
Picazo [82]	2008	2009	Spain	703	520	201	183	5	5		BMD	Blood
Jones [63]	2007	2008	USA	4338	3318	1930	1020	2	2		BMD	Medical canters
Pfaller [80]	2002–2005	2010	USA	13,053	10,917	4947	2136			1	BMD	Medical canters
John [58]		2002	Canada	658			658			15	Agar dilution	Patient in hospitals
Sader [103]	2002–2004	2006	France	1479	1100		379	16		7	BMD	Skin infection, Blood
Sader [103]	2002–2004	2006	Greece	185	128		57			2	BMD	Skin infection, Blood
Sader [103]	2002–2004	2006	Turkey	462	291		171			2	BMD	Skin infection, Blood
Khan [66]	2012–2013	2014	Saudi Arabia	190			190			4	Microscan Walk Away system (40si, siemens)	Blood

Abbreviations: DD; disk diffusion, BMD; broth microdilution

**Table 4:** Characteristics of the articles that were included in the meta-analysis and reported resistance to daptomycin

First name	Time of study	Published time	Country	Total staphylococcus	*S. aureus*	MRSA	CoNS	*S. aureus* Daptomycin-Resistant	MRSA Daptomycin - Resistant	CoNS Daptomycin-Resistant	Susceptibility testing method	Isolation source
Morrissey [76]	2011	2012	Italy	82	41	41	41	3	3	1	BMD	Bacteraemia
Mendes [75]	2007–2009	2010	USA	4077	4077	4077		6	6		BMD	Bacteraemia, Pneumonia
Biedenbach [22]	2003–2004	2007	Australia	1559	1257	480	302	1	1		BMD	Skin, Blood, Respiratory tract infection
Picazo [84]	2001–2010	2011	Spain	1130	1130	1130		1	1		BMD	Medical canters
Picazo [83]	2001–2006	2010	Spain	1186	755	755	431	1	1		BMD	Blood
Vamsimohan [109]	2011	2014	India	50	50	30		2	2		E-test	Wound, Pus swab
Pfaller [80]	2002–2005	2010	USA	13,053	10,917	4947	2136	5	2	4	BMD	Medical centers
Jevitt [57]	1996–2001	2003	USA	119	88	47	31	3	3	2	BMD	Medical centers
Rouse [91]	1985–2005	2007	USA	184	68	68	116	2	2		BMD	Endocarditis, Joint infection
Rolston [88]	2011	2013	USA	165	106	72	59	1	1	3	E-test	Surgical wounds, Pleural, Ascitic fluid
Cuny [32]	2011–2013	2015	Germany	1952	1952	1952		7	7		BMD	Blood
Sader [100]	2007–2008	2009	USA	9230	8077	4514	1153	8	8	6	BMD	Blood, Skin, Pneumonia
Kao [64]	2006–2008	2011	Taiwan	470	470	470		2	2		BMD	Blood
Jain [54]	2011–2012	2013	India	73	68	31	5	3	3		E-test	Soft tissue, Blood, Intra-abdominal infection
Jones [63]	2007	2008	USA	4338	3318	1930	1020	4	3	4	BMD	Medical centers
Jones [60]	2006	2007	USA	3721	2913	1648	808	3	3		BMD	Pneumonia, Wound, Urinary tract
Sader [102]	2005–2010	2011	USA	22,858	22,858	12,181		13	12		BMD	Blood
Flamm [40]	2010	2012	USA	4049	3105	1578	944	5	5		BMD	Blood, Pneumonia, Skin
Farrell [38]	2008	2009	USA	4012	3156	1752	856	3	3	6	BMD	Pneumonia, Wound, Urinary tract
Flamm [41]	2013	2015	USA	3433	3035	1454	398	1	1		BMD	Blood, Skin, Soft tissue
Karlowsky [65]	2011–2015	2017	Canada	3760	3408	728		1	1		BMD	Urine, Blood, Respiratory tract, Skin, Wound, Body fluid
Sader [94]	2009–2013	2015	USA	4426	4426	2013		7	7		BMD	Blood
Chen [30]	2006–2010	2014	Taiwan	1725	1725	1725		2	2		BMD	Blood, Pus
Mendes [74]	2007–2009	2012	USA	9282	8042	4278	1240	8	8	3	BMD	Bacteraemia, Respiratory tract
Richter [87]	2009	2011	USA	4210	4210	2247		10	9		BMD	Wound Blood, Lower respiratory tract, and Joint fluid.
Biswas [23]	2010	2012	India	115	80	80	35	5	5		E-test	Abscesses, Wound, Skin
Morrissey [76]	2011	2012	Germany	81	43	43	38			3	BMD	Bacteraemia
Hellmark [50]	1993–2003	2009	Sweden	33			33			1	E-test	Infected Hip prostheses
Khan [66]	2012–2013	2014	Saudia Arabia	190			190			3	Microscan Walk Away system(40si,siemens)	Blood
Picazo [84]	2010	2011	Spain	702	503	187	199	1		1	BMD	Medical centers
Isnard [53]	2011–2014	2018	France	200	100	19	100			1	BMD	Prosthetic joint infections
Sader [99]	2003	2005	Latin America	787	536	143	251			1	BMD	Medical centers
Mathai [71]	2006	2007	India	1111	741	335	370	1		1	BMD	Medical centers
Sader [97]	2002–2006	2008	USA	8027	6497	3143	1530	1		4	BMD	Blood
Draghi [35]	2004–2005	2008	USA	2671	2299	1082	372	4		2	BMD	Medical centers
Stuart [107]		2011	Canada	633			633			7	Agar dilution method	Clinical isolates
Gales [45]	2005–2008	2009	Brazil	3030	2218	687	812			2	BMD	Blood, Skin, Pneumonia
Gallon [46]	2006–2007	2009	France	498			53			1	E-test	Abscess, whitlows, diabetic foot infections, impetigo, Furunculosis, wounds infections, cellulite, etc.
Zhanel [116]	2005–2006	2008	Canada	1046			162			2	BMD	Blood, urine, wound/tissue, respiratory specimens
Sader [104]	2005	2007	Italy	422			182			1	BMD	Blood, Skin, Pneumonia

Abbreviations: BMD; broth microdilution

**Table 5:** Characteristics of the articles that were included in the meta-analysis and reported resistance to linezolid

First name	Time of study	Published time	Country	Total staphylococcus	*S. aureus*	MRSA	CoNS	*S. aureus* Linezolid-Resistant	MRSA Linezolid - Resistant	CoNS Linezolid-Resistant	Susceptibility testing method	Isolation source
Mendes [75]	2007–2009	2010	USA	4077	4077	4077		5	5		BMD	Bacteraemia, Pneumonia
Cassettari [28]	2010–2011	2011	Spain	299	237	113	62	1	1	1	BMD	Skin and soft tissue infections, hospital-acquired pneumonia
Jain [55]	2011–2014	2015	India	2008	2008	384		3	3		E-test	
Duncan [37]	2013–2014	2016	USA	1353	1353	676		1	1		BMD	Pneumonia
Farrell [39]	2008–209	2011	USA	4073	3257	1673	816	5	5	12	BMD	Bacteraemia, Pneumonias, Wound infection, Pneumonia
Błażewicz [24]	2014–2015	2016	Poland	157	157	11		3			BMD	Skin, Nasal swab
Picazo [85]	2010	2011	Spain	702	503	187	199	2	2	16	BMD	Medical centers
Sader [92]	2005–2009	2010	brazil	2637	2637	846		2			BMD	Medical centers
Jevitt [57]	1996–2001	2003	USA	119	88	47	31	1			BMD	Medical centers
Cuny [32]	2011–2013	2015	Germany	1952	1952	1952		1	1		BMD	Blood
Sader [103]	2002–2004	2006	Greece	185	128		57	1			BMD	Skin infection, Blood
Campanile [27]	2012	2015	Italy	1684	1684	640		5	3		Automated VITEK-2 system, Broth microdilution	Lower respiratory tract, Skin and Soft tissue
Picazo [82]	2008	2009	Spain	703	520	201	183	6	6	3	BMD	Blood
Sader [100]	2007–2008	2009	USA	9230	8077	4514	1153	4	4	20	BMD	Blood, Skin, Pneumonia
Fuchs [44]	2000–2002	2002	USA	108	53	28	55	1	1		BMD	Medical centers
Sader [97]	2002–2006	2008	USA	6497	6497	3143	1530	6		6	BMD	Blood
Farrell [38]	2008	2009	USA	4012	3156	1752	856	3	3	18	Broth microdilution, E-test	Pneumonia, Wound, Urinary tract
Jones [61]	2007	2009	Ireland	141	130		11	1			BMD	Blood
Jones [63]	2007	2008	USA	4338	3318	1930	1920	2	2	18	BMD	Medical centers
Jones [60]	2006	2007	USA	3721	2913	1648	808	1	1	13	BMD	Pneumonia, Wound, Urinary tract
Ross [90]	2002	2005	USA	4557	3687	1401	870	1	1	1	BMD	Medical centers
Mendes [73]	2002–2004	2008	USA	1989	1989	1989		1	1		BMD	Medical centers
Flamm [40]	2010	2012	USA	4049	3105	1578	944	2	2	14	BMD	Blood, Pneumonia, Skin
Flamm [40]	2013	2015	USA	3433	3035	1454	398	2	2	3	BMD	Blood, skin, soft tissue
Putnam [86]	2004–2008	2010	USA	18,917	18,917	10,242		3	3		BMD	Bacteraemia, Pneumonia
Pfaller [81]	2011–2015	2017	USA	6741	3031	1391	924	1	1	7	BMD	Medical centers
Flamm [42]	2014	2016	USA	3903	3106		797	2	2	5	BMD	Blood, Pneumonia, Skin
Sahm [105]	2011–2012	2015	USA	4186	3743		443	5		2	BMD	Medical centers
Tekin [108]	2007–2011	2014	Turkey	870	90		771	1	1	14	E-test	Blood
Rosenthal [89]	2012	2014	Haiti	16	16	4		1			E-test	Different ward of hospital
Decousser [33]	2004–2016	2018	France	3437	3437	953		3			BMD	All body sites
Sader [96]	2008–2014	2016	USA	670	670	339		2	1		BMD	Skin infection
Hodille [51]	2010–2014	2016	France	440	440	325		2	2		BMD	Bronchopulmonary infections
Sader [94]	2009–2013	2015	USA	4426	4426	2013		1	1		BMD	Blood
Sader [95]	2008–2011	2013	USA	22,620	19,350	9872	3270	14	9	51	BMD	Blood, Respiratory tract, Skin
Mendes [74]	2007–2009	2012	USA	9282	8042	4278	1240	4	4	19	BMD	Bacteraemia, Respiratory tract
Richter [87]	2009	2011	USA	4210	4210	2247		1	1		BMD	Wound, Blood, Lower respiratory tract, Joint fluid
Gales [45]	2005–2008	2009	Brazil	3030	2218	687	812	1	1	2	BMD	Blood, Skin, Pneumonia
Sader [98]	2002–2006	2009	USA	14,009	14,009			2			BMD	Catheter related bloodstream infections (BSI)
Castanheira [29]	2006	2008	North America	4873	4288	2251	585	1	1	4	BMD	Bloodstream infections, Skin and soft tissue infections, Pneumonia
Morrissey [76]	2011	2012	Italy	82	41	41	41			2	BMD	Bacteraemia
Morrissey [76]	2011	2012	Spain	79	45	45	34			1		Bacteraemia
Cui [31]	2009–2010	2013	China	713			713			4	Agar dilution	Blood
Song [106]	2013–2014	2017	China	1104			1104			3	Agar dilution	Blood
Pedroso [78]	2008–2009	2018	Brazil	58			58			1	Automated VITEK-2 system	Blood
Li [67]	2014	2016	China	1798	1499	632	299			2	BMD	Pneumonia, Skin and soft tissue infection, Blood infection
Isnard [53]	2011–2014	2018	France	200	100	19	100			2	BMD	Prosthetic joint infections
Gandra [47]	2008–2014	2016	India	5426	1089	608	4337	17		21	BMD	Blood
Draghi [36]	2004	2005	USA	3368	2872	1556	496			1	BMD	Skin, Blood, Respiratory tract
Jones [61]	2007	2009	Italy	151	98		53			2	BMD	Blood
Mutnick [77]	2001–2002	2003	USA	5848	4677		1171			1	BMD	Blood, Skin, Respiratory and Urinary tract
Jones [62]	2008	2009	Italy	128	59		69			2	BMD	Medical centers
Jones [62]	2008	2009	France	140	100		40			1	BMD	Medical centers
Martinez [70]	2006	2013	Mexico	142			142			5	BMD	Blood
Zhanel [116]	2005–2006	2008	Canada	1046			162			2		Blood, urine, Wound/tissue, Respiratory specimens

Abbreviations: BMD; broth microdilution

## Discussion

MRSA is a frequent cause of skin and soft tissue infection, pneumonia, endocarditis, bone and joint infection in individuals with some risk factors such as indwelling devices, surgical interventions, long-term antibiotic use, intensive care admission, and dialysis [121, 122]. In recent years, this bacterium has had very high health costs for patients due to increased length of hospital stay and longer duration of antibiotic treatment [123]. Moreover, CoNS are opportunistic pathogens that lead to 30% of hospital-induced infections and 10% of uncomplicated urinary tract infections in young women and native valve endocarditis, especially in immunocompromised patients [124, 125]. Currently, the treatment of MRSA and CoNS is difficult due to the high antibiotic resistance to beta-lactams and other antibiotic classes, and newer agents such as linezolid, daptomycin, Q/D, and tigecycline can be used as alternative if available and deemed cost-effective. Accordingly, this study collected data from resistance to these antibiotics all over the world to determine the extent of their clinical application. The analysis of the results showed that linezolid had the highest inhibitory effect on *S. aureus*; due to the high volume of the samples in the studies and a small number of bacteria that have been reported as resistant (mostly in the United States), in terms of statistical analyses, the percentage of resistance to this antibiotic is zero (Table 1). It should be noted that the studies (20 studies) that used the DD method as an antibiotic susceptibility test for linezolid were removed from this study and not entered into statistical analyses. Furthermore, the most linezolid-resistance *S. aureus* isolates isolated from pneumonia and blood infections were the highest in number. In addition to the good effect of linezolid on *S. aureus*, this drug also had the efficient activity against MRSA, while the resistance of CoNS was higher to this antibiotic. One of the reasons for the increased resistance in CoNS is the ability of these bacteria to develop resistance quite easily following linezolid exposure, even though this has not been proven in vitro, to the best of our knowledge. Furthermore, more Linezolid-resistant CoNS (LRCoNS) were associated with outbreaks; 50% of those studies that analysed LRCoNS involved clonal LRCoNS across one or more patients and facilities. The studies that used MLST for typing of resistant-linezolid CoNS, ST5, ST22 and for *S. aureus* ST228, ST8 and ST5 were reported to be more sequence types related to linezolid resistance [25, 67].

Tigecycline had the best effect (equal to linezolid) on MRSA, and very low resistance in *S. aureus* was observed; however, CoNS with 1.6% showed the highest percentage of resistance to this antibiotic (Table 1). Since very few studies have reported the resistance of CoNS to tigecycline (Fig. 3), the high percentage of resistance noted by tigecycline cannot be deemed. The geographic diversity of the countries that reported the tigecycline resistance was higher than those with linezolid, thus showing more use of this antibiotic in different parts of the world. Recent MRSA infection treatment guidelines have not incorporated tigecycline. The reason is the FDA’s September 2010 safety statement, which describes increased overall mortality among severely infected patients who are treated with tigecycline; besides, cause of the excess deaths in these trials usually remains uncertain. However, it is likely that most cases of death among such patients were associated with the infection progression. Moreover, this antibiotic is not authorized for pneumonia or diabetic foot infections. Although tigecycline is recommended for treating skin and soft tissue infections, previous studies have shown no significant difference between this antibiotic and other new drugs, and tigecycline is referred to as the second or third line of treatment for infections caused by MRSA [126, 127]. Therefore, although the present study showed that *S. aureus* resistance to tigecycline is low, the use of this drug still has limitations in treating staphylococcal infections. Daptomycin is another new drug used to treat infections caused by Gram-positive bacteria including MRSA and VRE. It kills microorganisms by rapid membrane depolymerisation, loss of membrane potential and disruption of DNA, as well as RNA and protein-synthesis [128]. The daptomycin resistance among staphylococcal strains has been reported from around the world, although there has been no resistance report from the African continent. The United States had the highest rate of resistance (42.5% of studies); India, Taiwan, and Saudi Arabia reported resistance to this antibiotic from the Asian continent, and most of the bacteria were isolated from wounds and blood infections. In the United States and Europe, daptomycin is used for treating skin and soft tissue infections, bacteraemia, and endocarditis caused by *S. aureus* [129]. Previous studies have reported that it is not very practical to use daptomycin for the treatment of pneumonia, because it is deactivated by pulmonary surfactants. Therefore, vancomycin and linezolid are recommended to treat pneumonia caused by MRSA [130]. Our results have shown that daptomycin has the best performance with linezolid regarding CoNS, indicating that this antibiotic can be used for a therapeutic approach to infections caused by these bacteria. Furthermore, the present study showed that resistance to daptomycin has been very low (0.1–0.3%); considering that this antibiotic shortens the duration of the treatment of soft-tissue infections due to MRSA compared to vancomycin [131], it can be used to a greater degree for treating the mentioned infections. However, spontaneous resistance to daptomycin seems to occur rarely [132], and vancomycin can also decrease the function of this drug [130, 133]. Therefore, it is possible to isolate daptomycin-resistant strains from the areas where this antibiotic is not even used, and physicians usually use alternative agents (linezolid and vancomycin) instead of daptomycin, which can be considered as a factor. Daptomycin can be one of the choices for treating staphylococci-induced infections if there is a strong possibility based on local microbiological data or recent treatment history of vancomycin in an infected patient with MIC of > 1 μg/mL.

Q/D comprises quinupristin and dalfopristin in a 30:70 ratio, which prevents protein synthesis in bacteria [134]. Studies have shown that Q/D with 0.7% has the highest resistance rate amongst MRSA strains (Table 1). Resistance reports were gathered from the continents of America, Asia, and Europe, although more studies have been carried out in European countries. This antibiotic is used for the treatment of VRE bloodstream infection and complicated skin and soft tissues infections caused by MRSA and *Streptococcus pyogenes*. However, the results of this study showed that Q/D had a weaker inhibitory effect than linezolid and daptomycin on *S. aureus*, MRSA, and CoNS (Table 1); on the other hand, it has significant side effects (myalgia, arthralgia, increased alkaline phosphatase, and nausea), high drug interactions, and treatment costs [135], which led to the limited use of this antibiotic. Therefore, it is better to use other new alternative antibiotics instead of Q/D for treating of staphylococcal infections. The present study showed that although linezolid, Q/D, daptomycin, and tigecycline are prescribed by clinicians for about 15 to 20 years, there is still very low resistance to these antibiotics around the world. On the other hand, with the increasing resistance of staphylococci to vancomycin and the high side effects of other drugs such as cotrimoxazole, it seems that these antibiotics have to be used more often in the future. The results of a recent study on the global prevalence of vancomycin-nonsusceptible MRSA showed that the prevalence of vancomycin-intermediate *S. aureus* (VISA) was 3.01% in 68,792 MRSA strains. Furthermore, the pooled prevalence of heterogeneous vancomycin-intermediate *S. aureus* (hVISA) was 6.05% and is highly dangerous, because these bacteria lead to higher rates of vancomycin treatment failure. It should be noted that this study reported that the rate of vancomycin-nonsusceptible MRSA has been increasing in recent years, and this is a danger to the international community [136]. It should be noted that, still, some diseases caused by Staphylococcus genus, such as pneumonia, are treated easier with older drugs, and more studies are needed to evaluate the effect of the newer agents. The higher rates of resistance to the mentioned antibiotics in the United States and European countries compared to other parts of the world do not imply higher resistance to these antibiotics in this areas and are related to microbial susceptibility testing programs that are regularly carried out in these countries, while there are no such reports in the African and Asian countries (may because of non-availability and elevated prices in these regions). Therefore, by performing such programs in other countries, the exact resistance rates of the staphylococcal strains to the newer Gram-positive cocci antibiotics can be determined.

## Conclusion

The present study shows that resistance to new agents is low in staphylococci and these antibiotics can still be used for treatment of staphylococcal infections in the world. It should be noted that the development of resistance to these antibiotics should be prevented by appropriate antibiotic resistance testing programs.

## Data Availability

All data were included.

## Abbreviations

MRCoNS: Methicillin-resistant coagulase-negative staphylococci
PBP2: Penicillin-Binding Protein 2
FDA: Food and Drug Administration
EMA: European Medicines Agency
VRE: Vancomycin resistant enterococci
MeSH: Medical subject heading
CLSI: Clinical and Laboratory Standards Institute
EUCAST: European Committee on Antimicrobial Susceptibility Testing
BMD: Broth microdilution
REM: Random effects model
FEM: Fixed effects model
